# Moving gapless indirect excitons in monolayer graphene

**DOI:** 10.1186/10.1186/1556-276X-7-599

**Published:** 2012-10-30

**Authors:** Mahmood Mahmoodian, Matvey Entin

**Affiliations:** 1 Institute of Semiconductor Physics, Siberian Branch, Russian Academy of Sciences, Novosibirsk, 630090, Russia

**Keywords:** Monolayer graphene, Exciton, Energy spectrum, Optical absorption, Specific heat, 71.35.-y; 73.22.Lp; 73.22.Pr; 78.67.Wj; 65.80.Ck

## Abstract

The existence of moving indirect excitons in monolayer graphene is theoretically evidenced in the envelope-function approximation. The excitons are formed from electrons and holes near the opposite conic points. The electron-hole binding is conditioned by the trigonal warping of the electron spectrum. It is stated that the exciton exists in some sectors of the exciton momentum space and has the strong trigonal warping of the spectrum.

## Background

An exciton is a usual two-particle state of semiconductors. The electron-hole attraction decreases the excitation energy compared to independent particles producing the bound states in the bandgap of a semiconductor. The absence of the gap makes this picture inapplicable to graphene, and the immobile exciton becomes impossible in a material with zero gap. However, at a finite total momentum, the gap opens that makes the binding of the moving pair allowable.

The purpose of the present paper is an envelope-approximation study of the possibility of the Wannier-Mott exciton formation near the conic point in a neutral graphene. In the present paper, we use the term ‘exciton’ in its direct meaning, unlike other papers where this term is referred to as many-body (‘excitonic’) effects [1,2], exciton insulator with full spectrum reconstruction, or exciton-like singularities originating from saddle points (van Hove singularity) of the single-particle spectrum [3]. On the contrary, our goal is the pair bound states of electrons and holes. There is a widely accepted opinion that zero gap in graphene forbids the Mott exciton states (see, e.g., [4]). This statement which is valid in the conic approximation proves to be incorrect beyond this approximation. Our aim is to demonstrate that the excitons exist if one takes the deviations from the conic spectrum into consideration.

## Methods

We consider the envelope tight-binding Hamiltonian of monolayer graphene as follows:

(1) $$H_{\text{ex}}=∊(p_{e})+∊(p_{h})+V(r_{e}-r_{h}),$$

where

(2) $$∊(p)=\gamma_{0}\sqrt{1+4\text{cos}\frac{ap_{x}}{2}\text{cos}\frac{\sqrt{3}ap_{y}}{2}+4\overset{2}{\cos}\frac{ap_{x}}{2}},$$

is the single-electron energy, *a *= 0.246 nm is the lattice constant, $\left(\hbar =1\right)$, *V*(**r **)= −*e*^2^/(*χr*) is the potential energy of the electron-hole interaction. The electron spectrum has conic points *ν***K**,*ν *= ±1, **K **= (4*Π*/3*a*,0), where *∊*(**p**)≈*s*|**p**−*ν***K**|, $\left(s=\gamma_{0}a\sqrt{3}/2\right)$ is the electron velocity in the conic approximation.

The electron and hole momenta **p**_*e*,*h*_can be expressed via pair **q**=**p**_*e*_ + **p**_*h*_and relative **p**=**p**_*e*_−**p**_*h*_ momenta. The momenta **p**_*e*,*h*_ can be situated near the same (*q *→ *k *≪ 2*K*) or near the opposite conic points (**q **= 2**K** + **k **,*k *≪ 2*K*).

We assumed that graphene is embedded into the insulator with a relatively large dielectric constant *χ* so that the effective dimensionless constant of interaction $\left(g=e^{2}/(\mathrm{s\chi \hbar})∼2/\chi \ll 1\right)$ and the many-body complications are inessential. In the conic approximation, the classical electron and hole with the same direction of momentum have the same velocities *s*. The interaction changes their momenta, but not their velocities. The two-particle Hamiltonian contains no terms quadratic in the component of the relative momentum **p** along **k**. In a quantum language, such attraction does not result in binding. Thus, the problem of binding demands accounting for the corrections to the conic spectrum.

Two kinds of excitons are potentially allowed in graphene: a direct exciton with *k *≪ 1/*a*(when the pair belongs to the same extremum) and an indirect exciton with **q **= 2**K** + **k**. Assuming *p*≪*k* (this results from the smallness of *g*), we get to the quadratic Hamiltonian

(3) $$H_{\text{ex}}=\text{sk}+\frac{p_{1}^{2}}{2m_{1}}+\frac{p_{2}^{2}}{2m_{2}}-\frac{e^{2}}{\text{χr}},$$

where the coordinate system with the basis vectors **e**_1_≡**k**/*k *and **e**_2_⊥**e**_1_ is chosen, **r **= (*x*_1_,*x*_2_). In the conic approximation, we have *m*_2_ = *k*/*s*, *m*_1_ = *∞*. Thus, this approximation is not sufficient to find *m*_1_. Beyond the conic approximation (but near the conic point), we should expand the spectrum (2) with respect to **k** up to the square terms, which results in the trigonal spectrum warping. As a result, we have for the indirect exciton,

(4) $$\begin{array}{ll} \frac{1}{m_{1}}=\nu \frac{\text{sa}}{4\sqrt{3}}\cos3\phi_{k}, & \; \end{array}$$

where *ϕ*_**k**_ is an angle between **k **and **K**.

The effective mass *m*_1_ ≫ *m*_2_is directly determined by the trigonal spectrum warping, and the large value of *m*_1_ follows from the warping smallness. The sign of *m*_1_is determined by *ν *cos3 *ϕ*_**k**_. If *ν *cos3 *ϕ*_**k **_> 0, electrons and holes tend to bind, or else to run away from each other. Thus, the binding of an indirect pair is permitted for *ν*cos3*ϕ*_**k**_>0. Apart from the conic point, this condition transforms to

(5) $$\begin{array}{lll} & (1+u+v_{-})<0\wedge (1+u+v_{+})<0\vee \; & \;\\ & (1+u+v_{-})<0\wedge (1+v_{-}+v_{+})<0\vee \;\\ & (1+u+v_{+})<0\wedge (1+v_{-}+v_{+})<0,\; & \; \end{array}$$

where $\left(u=\text{cos}ak_{x},\;\;\;v_{\pm }\text{=}\cos((k_{x}\pm \sqrt{3}k_{y})a/2).\right)$

To find the indirect exciton states analytically, we solved the Schrödinger equation with the Hamiltonian (3) using the large ratio of effective masses. This parameter can be utilized by the adiabatic approximation similar with the problem of molecular levels. Coordinates 1 and 2 play a role of heavy ‘ion’ and ‘electron’ coordinates. At the first stage, the ion term in the Hamiltonian is omitted, and the Schrödinger equation is solved with respect to the electron wave function at a fixed ion position. The resulting electron terms then are used to solve the ion equation. This gives the approximate ground level of exciton *ε*(**k**)=*sk*−*ε*_*ex*_(**k**), where the binding energy of the exciton is *ε*_*ex*_(**k**) = *Π*^−1^*skg*^2^ log^2^(*m*_1_/*m*_2_) (the coefficient 1/*Π*here is found by a variational method).

A similar reasoning for the direct exciton gives negative mass *m*_1_=−32/(*ksa*^2^(7−cos6*ϕ*_**k**_)). As a result, the direct exciton kinetic energy of the electron-hole relative motion is not positively determined and that means the impossibility of binding of electrons with holes from the same cone point.

## Results and discussion

**Figure 1:**
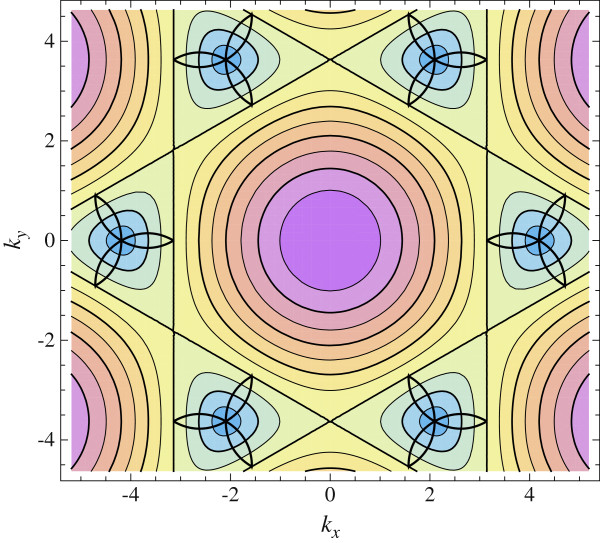
**Relief of the single-electron spectrum.** Domains where exciton states exist are bounded by a thick line.

Figure 1 shows the domain of indirect exciton existence in the momentum space. This domain covers a small part of the Brillouin zone.

The quantity *ε*_*ex*_(**k**) essentially depends on the momentum via the ratio of effective masses *m*_1_/*m*_2_. Within the accepted assumptions, *ε*_*ex*_ is less than the energy of unbound pair *sk*. However, at a small-enough dielectric constant *χ*, the ratio of both quantities is not too small. Although we have no right to consider the problem with a large *g* in the two-particle approach, it is obvious that the increase of the parameter *g* can only result in the binding energy growth.

**Figure 2:**
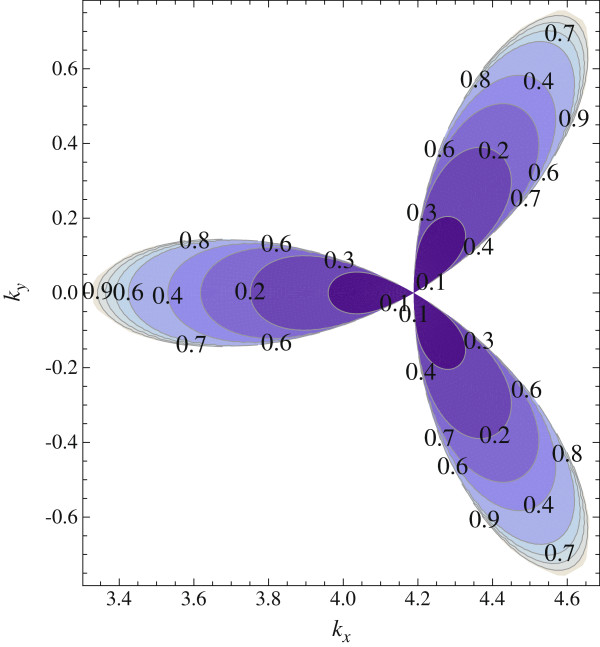
**Relief map of indirect exciton ground-state binding energy.** The map shows *ε*_*ex*_(in eV) as a function of the wave vector in units of reciprocal lattice constant. The exciton exists in the colored sectors.

**Figure 3:**
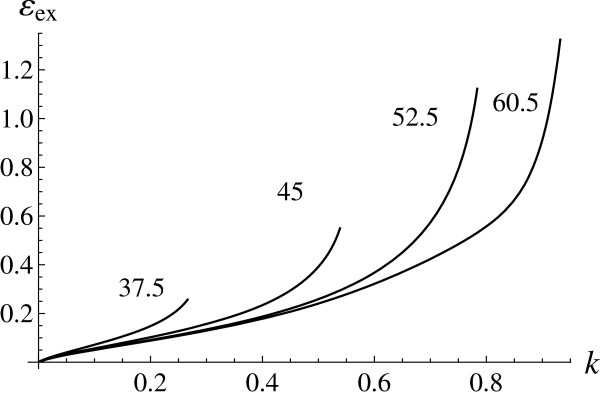
**Radial sections of Figure **2** at fixed angles in degrees (marked).** Curves run up to the ends of exciton spectrum.

Besides, we have studied the problem of the exciton numerically in the same approximation and by means of a variational approach. Figure 2 represents the dependence of the exciton binding energy on its momentum for *χ*=10. Figure 3 shows the radial sections of the two-dimensional plot. The characteristic exciton binding energies have the order of 0.2 eV.

All results for embedded graphene are applicable to the free-suspended layer if the interaction constant *g* is replaced with a smaller quantity $\left(\tilde{g}\right)$, which is renormalized by many-body effects. In this case, the exciton binding energy becomes essentially larger and comparable to kinetic energy *sk*.

We discuss the possibility of observation of the indirect excitons in graphene. As we saw, their energies are distributed between zero and some tenth of eV that smears up the exciton resonance. The large exciton momentum blocks both direct optical excitation and recombination. However, a slow recombination and an intervalley relaxation preserve the excitons (when generated someway) from recombination or the decay. On the other hand, the absence of a low-energy threshold results in the contribution of excitons in the specific heat and the thermal conductivity even at low temperature.

It is found that the exciton contribution to the specific heat at low temperatures in the Dirac point is proportional to (*gT*/*s*)^2^log^2^(*aT*/*s*)). It is essentially lower than the electron specific heat ∝(*T*/*s*)^2^ and the acoustic phonon contribution ∝(*T*/*c*)^2^, where *c* is the phonon velocity. Nevertheless, the exciton contribution to the electron-hole plasma specific heat is essential for experiments with hot electrons.

## Conclusions

In conclusion, the exciton states in graphene are gapless and possess strong angular dependence. This behavior coheres with the angular selectivity of the electron-hole scattering rate [5]. In our opinion, it is reasonable to observe the excitons by means of high-resolution electron energy loss spectroscopy of the free-suspended graphene in vacuum. Such energy and angle-resolving measurements can reproduce the indirect exciton spectrum.

## Competing interests

The authors declare that they have no competing interests.

## Authors’ contributions

All results were obtained by the collective work of MM and ME. Both authors read and approved the final manuscript.

## References

[B1] Yang L Deslippe J Park CH Cohen ML Louie SG Excitonic effects on the optical response of graphene and bilayer graphene Phys Rev Lett 2009 103 186802 19905823 10.1103/PhysRevLett.103.186802

[B2] Yang L Excitons in intrinsic and bilayer graphene Phys Rev B 2011 83 085405

[B3] Chae DH Utikal T Weisenburger S Giessen H vKlitzing K Lippitz M Smet JH Excitonic fano resonance in free-standing graphene Nano Lett 2011 11 1379 10.1021/nl200040q 21322607

[B4] Ratnikov PV Silin AP Size quantization in planar graphene-based heterostructures: pseudospin splitting, interface states, and excitons Zh Eksp Teor Fiz 2012 141 582 [JETP 2012, 114(3):512]

[B5] Golub LE Tarasenko SA Entin MV Magarill LI Valley separation in graphene by polarized light Phys Rev B 2011 84 195408

